# A neural network model was constructed by screening the potential biomarkers of aortic dissection based on genes associated with pyroptosis

**DOI:** 10.18632/aging.205187

**Published:** 2023-11-07

**Authors:** Cheng Chen, Lulu Gao, Hongwei Ge, Weibin Huang, Rong Zhao, Renjun Gu, Ziyun Li, Xin Wang

**Affiliations:** 1 Department of Vascular Surgery, The Third Affiliated Hospital of Soochow University, Changzhou, Jiangsu 213000, China; 2 Department of Anesthesiology, The Third Affiliated Hospital of Soochow University, Changzhou, Jiangsu 213000, China; 3 Department of Cardiology, The Third Affiliated Hospital of Soochow University, Changzhou, Jiangsu 213000, China; 4 School of Chinese Medicine and School of Integrated Chinese and Western Medicine, Nanjing University of Chinese Medicine, Nanjing, China; 5 Jinling Hospital, Affiliated Hospital of Medical School, Nanjing University, Nanjing, China; 6 School of Acupuncture and Tuina, School of Regimen and Rehabilitation, Nanjing University of Chinese Medicine, Nanjing, China

**Keywords:** aortic dissection, pyroptosis, neural network, biomarker, bioinformatics analysis

## Abstract

Background: Aortic dissection (AD) is one of the crucial and common cardiovascular diseases, and pyroptosis is a novel cell delivery mechanism that is probably involved in the pathogenesis of various cardiovascular diseases. However, no study has investigated the role of pyroptosis in AD.

Methods: We obtained two AD datasets, GSE153434 and GSE190635, from the Gene Expression Omnibus database. The differential expression of AD-related genes was determined by differential analysis, and their enrichment analysis was performed using Gene Ontology and Kyoto Encyclopedia of Genes and Genomes databases. Additionally, a protein–protein interaction network was established. Next, potential biomarkers were screened by Lasso regression analysis, and a neural network model was constructed. Finally, the potential biomarkers were validated by constructing a mouse model of AD.

Results: A total of 1033 differentially expressed related genes were distinguished and these genes were mainly associated with the phosphoinositide 3-kinase (PI3K)/protein kinase B (Akt) and mitogen-activated protein kinase signaling pathways. The Lasso regression results showed five potential biomarkers, namely platelet endothelial cell adhesion molecule-1 (PECAM1), caspase 4 (CASP4), mixed lineage kinase domain-like pseudokinase (MLKL), APAF1-interacting protein (APIP), and histone deacetylase 6 (HDAC6) and successfully constructed a neural network model to predict AD occurrence. The results showed that CASP4 and MLKL were highly expressed, whereas PECAM1 and HDAC6 were lowly expressed in AD samples, and no statistically significant difference was observed in APIP expression in AD samples.

Conclusion: Pyroptosis plays a crucial role in AD occurrence and development. Moreover, the five potential biomarkers identified in the present study can act as targets for the early diagnosis of AD in patients.

## INTRODUCTION

Aortic dissection (AD) is a high-risk cardiovascular disease [1], and a delay in its treatment can increase the mortality rate from 24% to 48% within a couple of hours [2]. Therefore, AD treatment is one of the most difficult procedures in vascular surgery. In recent years, the incidence of AD has gradually increased, and the trend is gradually younger [3]. According to the previous research, AD can be classified as Stanford type A and Stanford type B. The mortality rate of severe AD with aortic rupture is almost 90%. At present, the most effective strategy for acute aortic dissection is surgical treatment, but the technical requirements for surgical treatment are very high [4]. No effective medical treatment is available to control its progression to severe AD; thus, identifying the possible biomarkers of subatomic disease and severe AD is particularly important.

A study has shown that AD emergence and development may be related to inflammation [5]. Additionally, the genes of interleukin (IL)-6 and IL-8 were expressed in thoracic aortic dissection [6, 7], and the gene expression of IL-2, IL-6, and transforming growth factor-beta tended to be upregulated in patients with Stanford type A [8, 9]. Additionally, vascular inflammation is a risk factor for aortic wall damage. For example, monocytes and macrophages play an essential role in the immune system in AD development [4, 10].

Pyroptosis is a form of inflammation-induced cell death that causes cell swelling and febrile vesicle rupture after cell death [11]. Accumulating evidence has supported the association between pyroptosis and heart diseases. For example, in atherosclerosis, nucleotide-binding domain, leucine-rich–containing family, pyrin domain–containing-3 activates the NADPH oxidase-dependent pathway to induce reactive oxygen species production, thereby promoting pyroptosis [12].

No study has used the bioinformatics approach to investigate the association between pyroptosis and AD. In the present study, we performed a detailed bioinformatics analysis using the Gene Expression Omnibus (GEO) dataset to determine whether pyroptosis contributes to aortic development. Additionally, we established a neural network model to predict AD incidence and identify AD-associated pyroptosis biomarkers. To better elucidate the immunity-associated molecular mechanisms underlying AD development, we investigated the relationship between pyroptosis and immune infiltrating cells.

## MATERIALS AND METHODS

### Data sources and processing

AD cohort transcripts with matching clinical data were downloaded from the GEO database. After analysis, GSE153434 was selected as a training set and GSE190635 as a validation set.

### Screening for pyroptosis-related genes

We used the “limma” (v 3.48.3) R package to screen differentially expressed genes (DEGs) and presented the results in the form of log_2_FC > 0.6, *p* < 0.05. GeneCard was used to identify genes related to pyroptosis. A Venn diagram was used to identify six pyroptosis-related DEGs (1.7.1). Finally, these DEGs were presented in the form of heatmaps.

### Gene enrichment analysis between high-risk and low-risk groups

To reveal the effect of potential DEG-associated biological pathways, we used the R package clusterProfiler for Gene ontology (GO) enrichment analysis and Kyoto Encyclopedia of Genes and Genomes (KEGG) pathway analysis.

### Protein–protein interaction (PPI) network and identification of hub genes

The PPI network of the identified DEGs was visualized using the STRING database (https://string-db.org/) and Cytoscape software (version 3.8.2). The CytoHubba tool of the Cytoscape software was used to identify hub genes in the PPI network.

### Biomarker screening

We used the Lasso regression algorithm to screen potential biomarkers of AD. The accuracy of the obtained biomarkers was verified by the receiver operating characteristic (ROC) curve analysis. Next, the biomarkers were verified using an external validation set.

### Construction of a neural network diagnosis model

We constructed a neural network model of AD on the basis of the selected potential biomarkers using the R language packages “neuralnet” and “NeuralNetTools.” Further, model accuracy was verified by ROC curve analysis.

### Immunoinfiltration analysis

We used the CIBERSORT algorithm to evaluate 22 kinds of immune cells. The number of immune cell types was estimated for 1000 permutations using a reference system containing 22 isomers (LM22). “corrplot” in R was used to associate 22 types of infiltrating immune cells for correlation analysis.

### Construction of a mouse model of AD

Male C57 mice (22–24 g, 7–8 weeks old) were purchased from Jiangsu Co., Ltd., China. After a week of adaptation, the mice were divided into the normal control and AD groups, with six mice in each group. The AD group was fed with β-aminopropionitrile (BAPN) for three weeks.

After three weeks, micropumps containing angiotensin II (Ang-II) were implanted in the neck and back of the mice, and AD tissue samples were collected after three days of the sustained release of Ang-II. Normal saline was administered to the control group. All mice were killed by cervical dislocation. Aortic tissues were collected, and their length and thickness were measured.

### Quantitative reverse transcription polymerase chain reaction (qRT-PCR)

RNA was extracted using an RNA isolator (Guangzhou, China). Reverse transcription was performed using qPCR-based HiScript II QRT Super Mix (Nanjing, China). ChamQ Universal SYBR qPCR Master Mix (Nanjing, China) was used for mRNA detection according to the manufacturer’s instructions. The primer sequences used are listed in Supplementary Table 1. ACTB expression was used as a measure of mRNA expression by the 2–ΔΔCt method.

### Statistical analysis

The R 4.2.0 programming language was used to perform statistical analysis. Statistically significant differences between the two groups were determined by Spearman’s rank test. The data were presented as the mean ± standard deviation. Values at *p* < 0.05 were considered statistically significant.

### Data availability statement

The datasets presented in this study can be found in online repositories. More data are accessible in the supplementary documents.

## RESULTS

### Differential analysis

**Figure 1:**
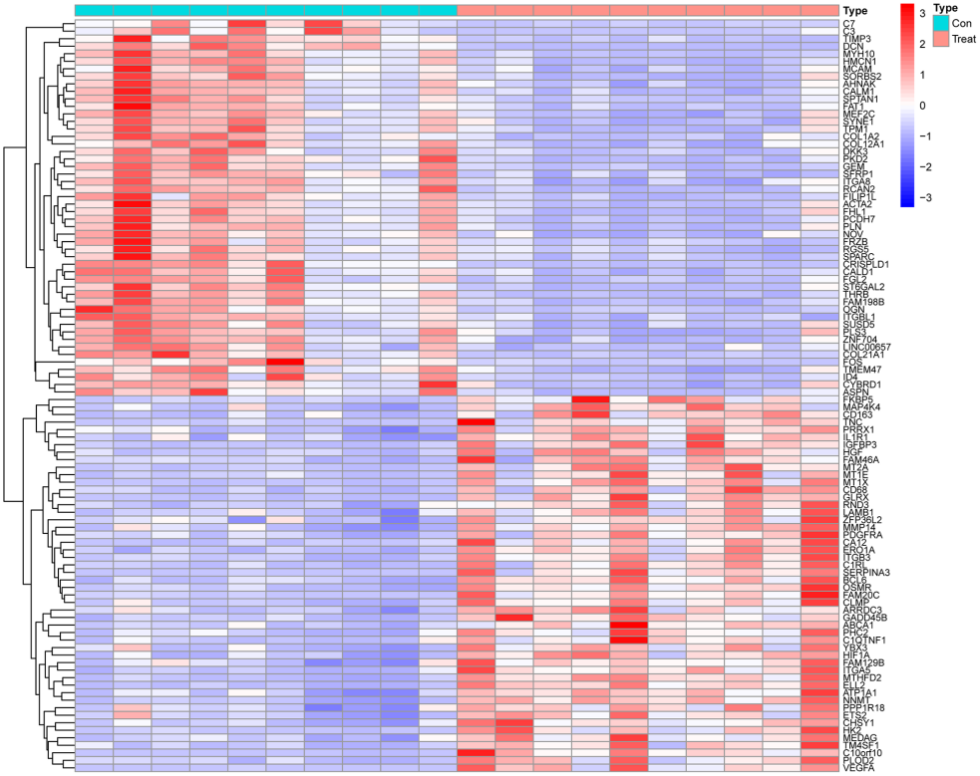
Heatmap of the top 50 differential genes.

We used “limma” package to identify DEGs between 10 patients with AD and 10 controls in GSE153434. We total identified 1033 AD-related DEGs. We visualized the top 50 differential genes (Figure 1).

### Gene enrichment analysis

**Figure 2:**
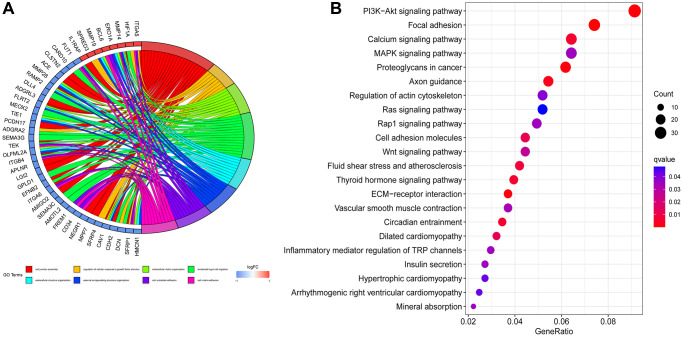
**Enrichment analysis.** (**A**) Gene Ontology enrichment analysis. (**B**) Kyoto Encyclopedia of Genes and Genomes enrichment analysis.

We analyzed the biological processes and signaling pathways of the identified differential genes. In our study results, the biological processes that were significantly enriched in AD patients were cell junction assembly, regulation of cellular response to growth factor stimulus, extracellular matrix organization, ameboidal−type cell migration, extracellular structure organization, external encapsulating structure organization, cell−substrate adhesion, cell−matrix adhesion (Figure 2A). In addition, the phosphoinositide 3-kinase (PI3K)/protein kinase B (Akt) signaling pathway, Focal adhesion, Calcium signaling pathway, MAPK signaling pathway, Proteoglycans in cancer, Axon guidance signaling pathway are a significantly enriched signaling pathway for AD (Figure 2B).

### PPI network analysis

**Figure 3:**
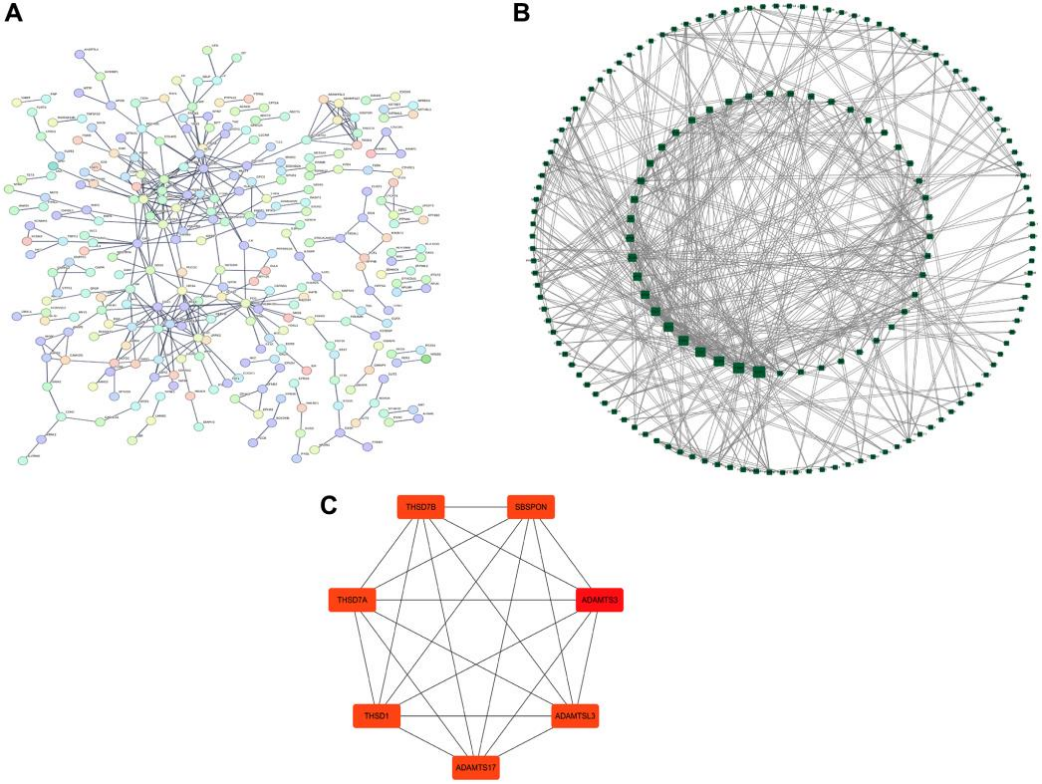
**Protein–protein interaction (PPI) network analysis.** (**A**) PPI network. (**B**) Visual analysis using Cytoscape. (**C**) Hub gene screening.

The STRING database was used to establish a PPI network of DEGs (Figure 3A), and the Cytoscape software was used to visualize the PPI network and analyze hub genes (Figure 3B). The CytoHubba MCC module of the Cytoscape software showed the top seven hub genes, namely THSD7B, SBSPON, THSD7A, ADAMTS3, THSD1, ADAMTSL3, and ADAMTS17 (Figure 3C). In the map of hub gene building blocks, ADAMTSL3, ADAMTS17, and THSD1 occupied the main positions.

### Screening of potential biomarkers

**Figure 4:**
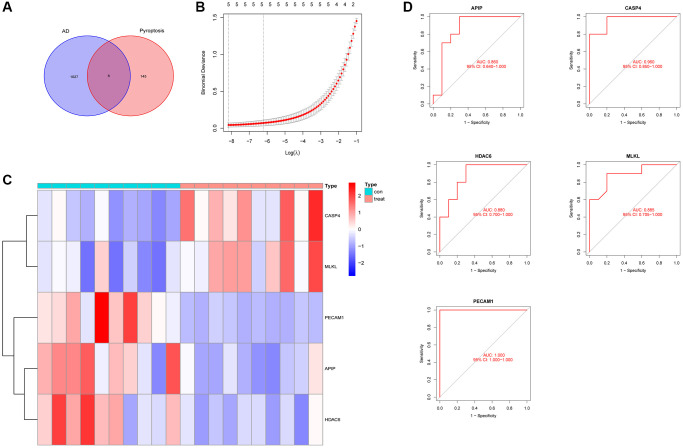
**Screening of potential biomarkers.** (**A**) Differential gene screening related to pyroptosis. (**B**) Lasso regression screening for potential markers. (**C**) Heatmaps of five potential biomarkers. (**D**) Receiver operating characteristic curves for five potential biomarkers.

**Figure 5:**
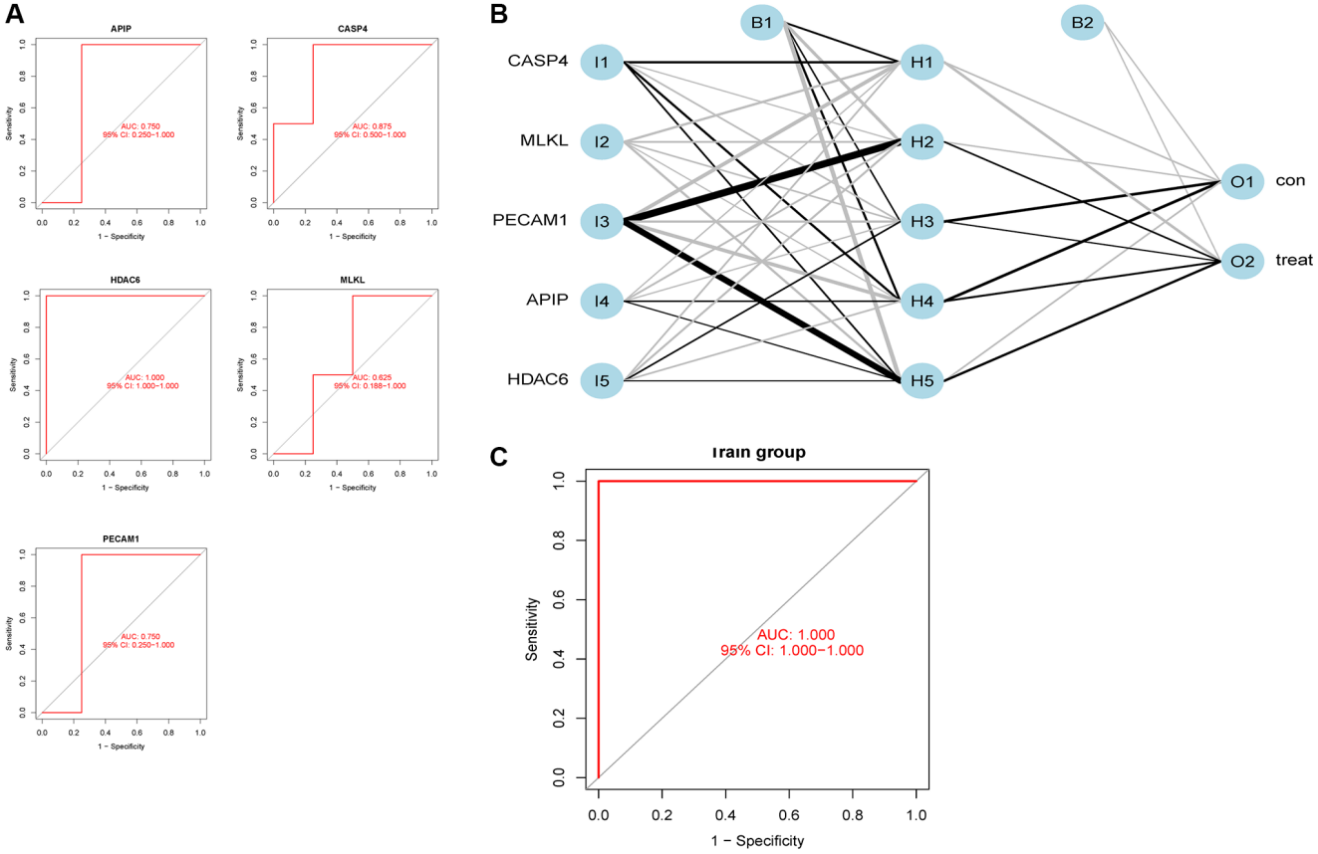
**Neural network construction.** (**A**) External dataset validation of five potential biomarkers. (**B**) Neural network model construction. (**C**) Receiver operating characteristic curve of the neural network model.

We obtained 151 pyroptosis-related gene sets from GeneCard and six pyroptosis-related DEGs from the Venn diagram (Figure 4A). We screened these six DEGs and obtained five potential AD-related biomarkers, namely platelet endothelial cell adhesion molecule-1 (PECAM1), caspase 4 (CASP4), mixed lineage kinase domain-like pseudokinase (MLKL), APAF1-interacting protein (APIP), and histone deacetylase 6 (HDAC6), using the Lasso regression algorithm (Figure 4B). The heatmaps of the differential expression of these biomarkers are shown in Figure 4C. We verified the accuracy of the biomarkers by performing ROC curve analysis (Figure 4D), and the areas under the curve (AUCs) of the five biomarkers were 1.000, 0.960, 0.885, 0.860, and 0.880, respectively. Additionally, their accuracy was verified using an external validation set with AUCs of 0.750, 0.875, 0.625, 0.750, and 1.000, respectively (Figure 5A).

### Neural network model construction

We successfully constructed a neural network model to predict AD occurrence by screening the five potential biomarkers using “neuralnet” and “NeuralNetTools.” The outcome index was AD and control (Figure 5B). The ROC curve analysis performed to verify the accuracy of the model showed an AUC of 1.000 (Figure 5C).

### Immune cell infiltration

**Figure 6:**
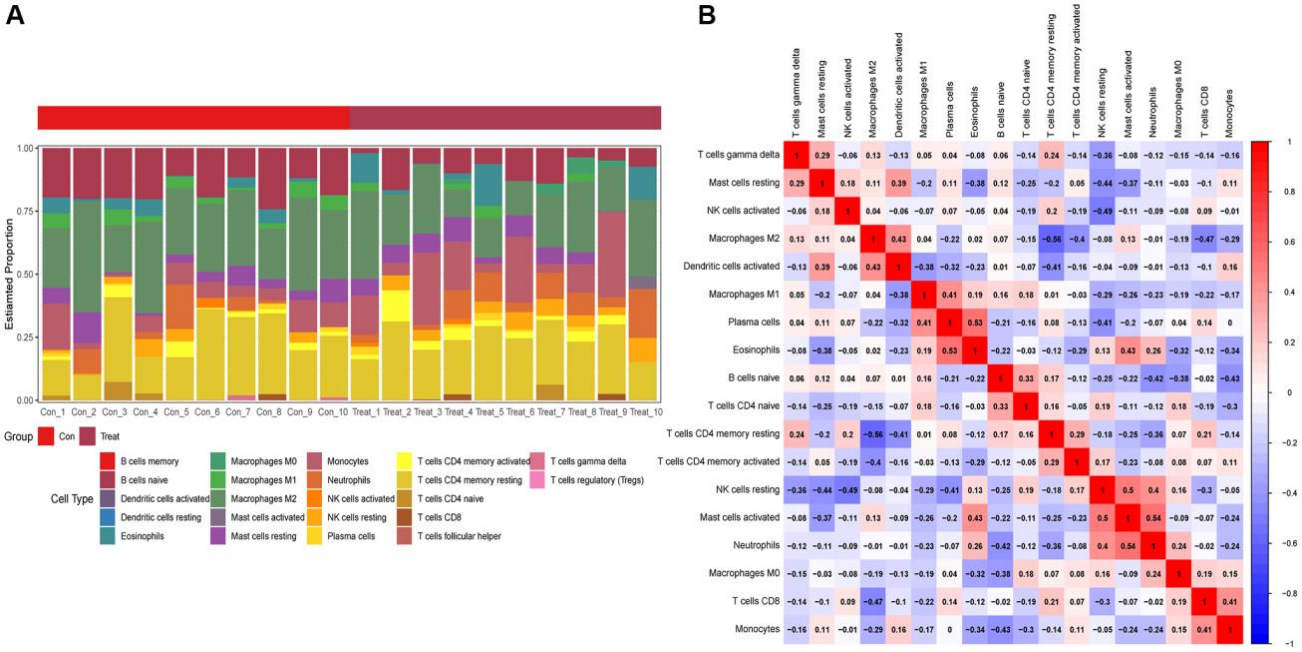
**Immunoenrichment analysis.** (**A**) Heatmap of immune-cell distribution in aortic dissection tissues. (**B**) Immune cell correlation analysis.

Based on the examination results, the safe framework was planned (Figure 6A), and the first immunological highlights of promotion were found. A correlation analysis was performed to understand interactions between immune-system cells. Figure 6B shows a strong correlation between the combined data and the immune system.

### Validation of potential biomarkers

**Figure 7:**
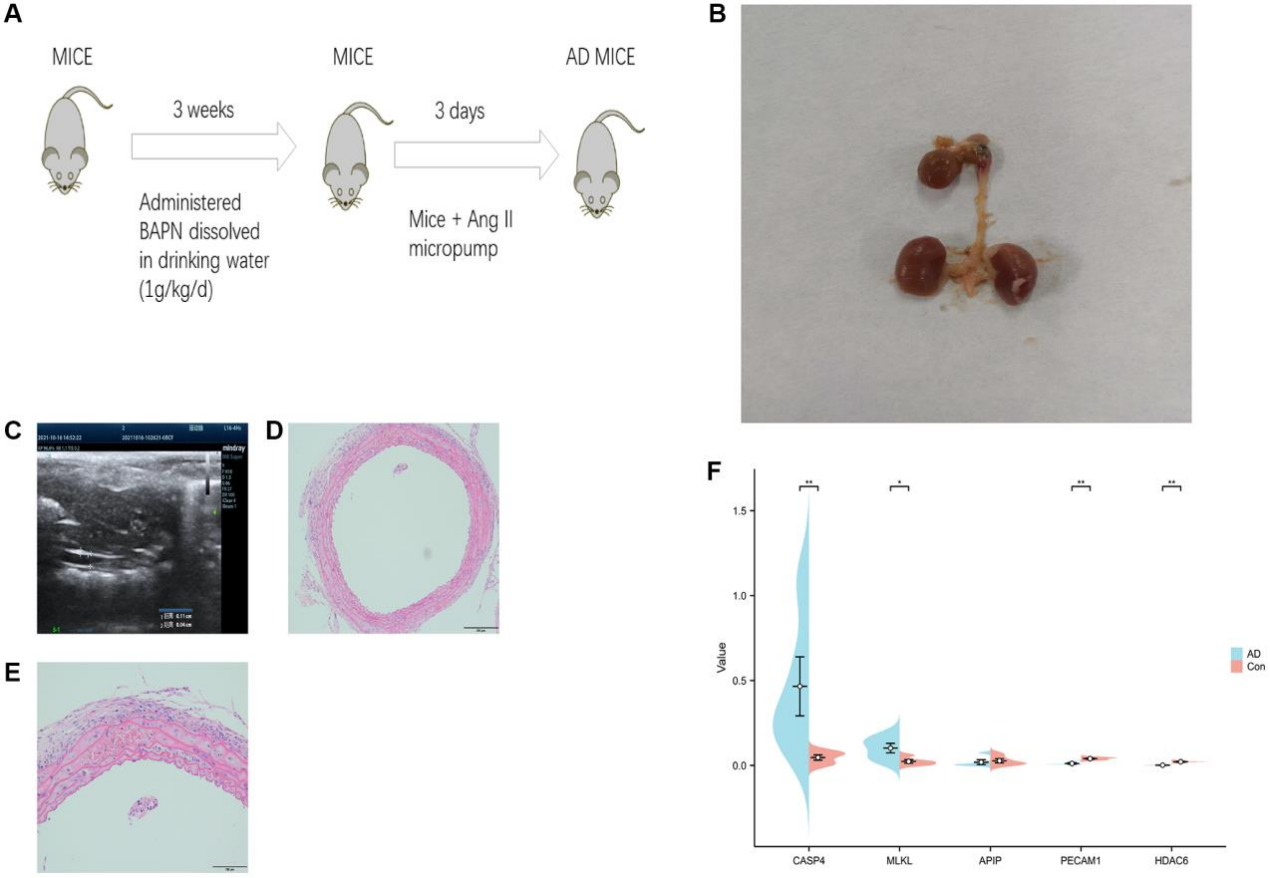
**Potential biomarkers were validated in mouse models of aortic dissection (AD).** (**A**) Flow chart of the AD mouse model. (**B**) AD, (**C**) B-ultrasound of the AD mouse model. (**D**, **E**) Hematoxylin/eosin staining of the AD mouse model. (**F**) Polymerase chain reaction analysis of five potential biomarkers. ^*^p < 0.05, ^**^p < 0.01.

The mouse AD model was established using the combination of BAPN and Ang-II. After feeding one gram of β-BAPN per kilogram of body weight per day for three weeks, a micropump containing Ang-II was implanted subdued into the neck and back of mice and released continuously for 3 days to successfully establish an AD model. The evaluation value of B-ultrasonography of AD in the mice was observed using an ultrasound imaging system according to a histopathological control study (Figure 7A). The false lumen of the blood vessel was observed by B-ultrasound. The hematoxylin/eosin staining showed that the structure of the blood vessel wall was damaged, the annulus fibrosus was broken, the false lumen was sandwiched, and the false lumen was filled with several red blood cells and infiltrated inflammatory cells (Figure 7B–7E). Further, we detected the expression of the biomarkers in the model samples by PCR (Figure 7F), and the results showed that CASP4 and MLKL were highly expressed in the AD samples, whereas PECAM1 and HDAC6 were lowly expressed in the AD samples. No statistically significant difference was observed in APIP expression in the AD samples.

## DISCUSSION

AD is a fatal disease. Studies have shown that the mortality rate of patients with acute aortic dissection within 48 hours of onset increases by 2% every 2 hours [13], and the mortality rate of patients with acute aortic dissection who have undergone surgical treatment is around 10% [14]. For patients with acute aortic dissection, the efficacy of traditional drug treatment is limited, while surgical treatment is very difficult, with high technical requirements and high mortality. Therefore, it is necessary to find a novel and effective biomarker identification to predict the occurrence and development of diseases, so as to achieve early diagnosis and timely treatment, and improve the survival rate of patients.

In a previous study [15], the inflammation index was considered a reliable indicator to predict the short-term survival rate of patients with AD after surgery, and inflammation is a probable risk factor for aortic wall thinning in patients with acute AD [6]. Although numerous pathological mechanisms underlie AD development, underlying molecular pathways are difficult to identify.

Herein, we performed differential analysis on the datasets selected from the GEO database. Subsequently, we performed GO and KEGG enrichment analyses on the obtained DEGs and found that the enrichment pathway was mainly related to inflammation. For instance, during myocardial infarction, PI3K/Akt, a regulator of angiogenesis, was involved in the proliferation and apoptosis of cardiomyocytes, fibroblasts, and monocytes via inflammation [16]. A previous study showed that the PI3K/Akt signaling pathway improved neuroinflammatory responses and cognitive impairment in Alzheimer’s disease mice [17]. Ma reported that baicalin inhibited the activation of the pro-inflammatory MAPK signaling pathway by increasing the level of renal glutathione peroxidase, thus inhibiting the infiltration of inflammatory cells [18].

We further identified five pyroptosis-related genes that may serve as potential diagnostic biomarkers for AD. PECAM 1 was first reviewed in detail by Newman [19], and they emphasized its role as an adhesion receptor in thrombus formation, hemostasis, immunity, and inflammation. Bayat et al. [20] found that antibodies with Pecam1 gene deletion led to neutrophil accumulation between endothelial cells and basement membrane *in vivo*. Recent studies have shown that neutrophil count is related to cardiovascular disease occurrence and development and possesses important predictive value for various cardiovascular diseases [21, 22]. These findings provide strong evidence for the role of PECAM 1 as a prognostic marker for cardiovascular diseases.

As a receptor of cytoplasmic lipopolysaccharides, CASP4 is involved in diverse inflammation-related reactions and can induce pyroptosis and IL-18 production, thus further aggravating inflammatory damage [23]. CASP4 regulates inflammatory responses [24]. In the case of a persistent or excessive inflammatory response, macrophages penetrate the damaged endothelial wall, phagocytose abnormal cholesterol on the surface, and promote plate formation [25].

Sun et al. [26] proposed that MLKL can inhibit tumor necrosis factor-induced necrosis and is regulated by type I/II interferons, inflammatory conditions, and tissue damage. MLKL has been identified as a downstream target of receptor-interacting protein kinase-3 and an end effector of necrosis [27–29].

Cho and colleagues [30] believed that APIP affected cytochrome c release by inhibiting caspase-3/9 activity, thereby inhibiting cell apoptosis. A study on heart disease showed that APIP was correlated with ADORA2B mRNA levels. Additionally, the records of both groups were higher in the hearts of patients with heart failure than in controls. A few more studies showed that APIP expression in neonatal primary cardiomyocytes was significantly upregulated under hypoxic conditions [31, 32]. These results suggested that APIP could be used as a diagnostic biomarker in order to establish treatment guidelines for cardiovascular diseases, such as AD.

HDAC6 plays a vital role in maintaining ventricular muscle stiffness. Lin et al. [33] found that HADC6 expression inhibition increased myofibril stiffness. Furthermore, diastolic dysfunction was caused by the loss of HADC6. Additionally, similar to CASP4, HADC6 was involved in inflammation activation by affecting cell apoptosis activation [34, 35].

This study contains some limitations. The sample size of the dataset we used was too small, which led to some bias in the results. In addition, we did not explore the mechanism of the potential biomarkers we screened.

## CONCLUSION

The present findings revealed the critical role of pyroptosis in AD occurrence and development. Moreover, five potential biomarkers suitable for the early diagnosis of AD were identified. Furthermore, these biomarkers could be considered potential treatment targets for AD. Additionally, a neural network model was built on the basis of the biomarkers to predict the incidence of AD.

## Supplementary Materials

Supplementary Table 1
