# Supplementary material for: A neural network model was constructed by screening the potential biomarkers of aortic dissection based on genes associated with pyroptosis
Source: Aging (Albany NY). 2023 Nov 7;15(21):12388–99. doi: 10.18632/aging.205187 (PMC10683593; doi:10.18632/aging.205187)
Supplement: Supplementary Table 1 [file aging-15-205187-s001.pdf]

SUPPLEMENTARY TABLE

Supplementary Table 1. Gene qRT-PCR sequence.

CASP4-F	GTGGTGAAAGAGGAGCTTACAGC
CASP4-R	GCACCAGGAATGTGCTGTCTGA
MLKL-F	ATCAGCCGGACAGCAAAGAG
MLKL-R	GAATCACAGCCTTCAAATGGG
APIP-F	AAGGCAACTGCTGTCCTTGT
APIP-R	TTCAGCCTGTGTCTTGGCAT
PECAM1-F	ATGGAGCAGGACAGGTTCAGTC
PECAM1-R	AAGTGGAGTCCAGCCGCATATC
HDAC6-F	AAGTGGAAGAAGCCGTGCTA
HDAC6-R	ATCATAGACCAGCCCAGTGC
